# Biological, demographic, and health factors associated with HPV infection in Ecuadorian women

**DOI:** 10.3389/fpubh.2023.1158270

**Published:** 2023-06-15

**Authors:** Carlos Roman, Diego Andrade, Yenima Hernández, Zoila K. Salazar, Lizette Espinosa, Erika Campoverde, Lourdes Guallaizaca, María Merchán, Miriam Sarmiento, Jonathan Brenner

**Affiliations:** ^1^Diagnostic Department, MEDsan, Inc., Saint Petersburg, FL, United States; ^2^Investigation Center for Health, Academic Unit of Health and Wellness, Catholic University of Cuenca (UCACUE), Cuenca, Ecuador; ^3^Department of Mental Health, The Angels Mental Health Community, Tampa, FL, United States; ^4^Medical Center Association for the Well-being of the Ecuadorian Family (APROFE), Cuenca, Ecuador; ^5^Obstetrics Department, San Juan de Dios Hospital, Cuenca, Ecuador

**Keywords:** HPV, cervical cancer, sexually transmitted infection, risk factors, genotypes

## Abstract

**Objectives:**

The study aims to identify the correlation between human papillomavirus (HPV) infection and sociodemographic and sexual reproductive health factors in Ecuadorian women from March to August 2019.

**Methods:**

120 women were randomly selected from two gynecological clinics to complete a questionnaire and provide a biospecimen. PCR-Hybridization was used to genotype 37 HPV serotypes in samples obtained by endo-cervical brushing for liquid-based cytology. Sociodemographic and sexual health data were collected through a validated questionnaire during a medical consultation. Mathematical modeling of HPV infection was done using bivariate logistic regression.

**Results:**

65.0% of the women sampled had an HPV infection; 74.3% of these women had co-infections with other HPV genotypes. Out of the women who were HPV positive, 75.6% were diagnosed with high-risk genotypes from HPV strains 18, 35, 52, and 66. Parity, immunosuppression, and use of oral contraception/intrauterine devices (IUDs) were identified as associated variables. The explanatory model had a sensitivity of 89.5% and a specificity of 73.8%.

**Conclusion:**

The predominant strains of HPV among Ecuadorian women are diverse. The risk of HPV infection is a complex phenomenon where biological and psychosocial variables are integrated into a model. In populations with limited access to health services, low socioeconomic status, and negative sociocultural beliefs about sexually transmitted infections (STIs), surveys can be used as a pre-screening step for HPV infections. The diagnostic value of the model should be tested in multicenter studies that include women from all over the country.

## 1. Introduction

Human papillomavirus, the most common STI worldwide (1), is associated with genital, anal and oral cancers in men and women (2, 3). With over 200 HPV subtypes, at least 15 are considered to have a high oncogenic risk (4). Specifically, high-risk HPVs (hrHPV) are responsible for 70–90% of anogenital cancers in women (5). While HPV vaccination has greatly reduced the incidence rate and risk of mortality, the global burden of uterine cervical cancer (UCC) still exists; just shifted to countries with poor access to preventative measures. This creates a unique epidemiological pattern, where regions like Latin America are disproportionately affected. Currently, HPV is the leading cause of cervical cancer in the region with an incidence of infection twice as high as the global average (6).

There are a variety of risk factors associated with recurring HPV infections. Among these, early intercourse, number of sexual partners, and infrequent condom use are the most prevalent (7). Differences in prevalence due to sociodemographic factors including age, marital status, income level and race have also been documented (8). Additionally, health behaviors including poor diet, smoking (9), drug addiction, and inadequate hygiene contribute to the progression of HPV (10). Lack of information about HPV, inadequate access to testing, vaccination, and treatment have also been identified (11, 12).

Chronic infection of HPV strains 16 and 18 have been implicated in the majority of cervical cancer cases; a statistic that led to the creation of the HPV vaccinations, such as Gardasil and Cervarix (13). In Ecuador, while these vaccinations have substantially decreased HPV strains 16/18, the diversity of alternate high risk HPV strains has increased (14).

To reduce this incidence rate and premature mortality, this research aims to associate HPV infection in Ecuadorian women from Cuenca with sociodemographic, sexual-reproductive health and biological variables. Using this data, we aim to create a mathematical model that will aid in HPV pre-screening, contributing to Ecuador’s goal to reduce 25% of mortality from cancer by 2025 (15).

## 2. Materials and methods

### 2.1. Study design

This study includes a quantitative cross-sectional evaluation of women who attended two gynecology clinics in the city of Cuenca, Ecuador from March 2019 to August 2019.

The potential sample size was calculated using the sample size estimation formula for proportions in known populations plus 10% of estimated losses. The participating population size was estimated according to the volume of work activity of previous months in the gynecology service in that period. The rate of known HPV infection in the region was retrieved from previous studies.

The formula used is as follows:

$$n=\left(N*Z\propto^{2}*p*q\right)/\left[d^{2}*\left(N-1\right)+Z\propto^{2}*p*q\right]$$

Total sample was estimated to be 132 (119 + 13 possible lost patients) (population *N* = 175, significance level ∝ = 0.05, precision d = 5%, lost samples = 10%, HPV infection probability *p* = 40%).

Using random sampling, the patients were assigned sequential numbers upon arrival at the healthcare facility during the research period. Upon initial consultation, the medical professional assessed whether the participant met the following inclusion/exclusion criteria:

*Inclusion criteria:* current treatment at the respective healthcare facility; sexually active life; age between 18–65 years old; female.

*Exclusion criteria:* pregnancy; previous diagnosis of HPV infection or cervical cancer; treatment with immunosuppressive drugs.

The final sample in the study was 120 women.

### 2.2. Information collection

A patient questionnaire was used for the collection of sociodemographic and sexual-reproductive health data and was applied with reliability (Cronbach’s alpha = 0.79) and acceptable content validity (Aiken’s V of 0.82) (16). The questionnaire addressed age, place of residence, education level, personal income, alcohol and tobacco history, marital status, age of first coital intercourse, number of sexual partners, frequency of intercourse per month, access to gynecologic care, contraceptive use of condoms, IUDs or contraceptive pills, parity, history of STIs, and level of HPV awareness. Immunity was evaluated using 10 questions corresponding to symptoms of immunosuppression (tiredness/decay, allergies, eczema, skin abrasions, wounds that are slow to heal, loss of appetite, muscle pain, chills, headache, fever, respiratory infections, gastrointestinal infections, and sleepiness) (17) with a cutoff value over 5 points to be considered. The questionnaire was administered by a trained facilitator during medical consultation. The average response time was 14 min.

### 2.3. Biospecimen collection

The collection of biospecimens was carried out according to the protocols issued by the Ministry of Public Health (MSP) of Ecuador (15). Endocervical brushing was performed using SurePath® Liquid Based Cytology sampling kits according to manufacturer instructions [BD (18)]. The biological sample was taken by the gynecologist’s physician. The patient was in the supine ulnar position on the gynecological care table. The neck of the uterus was accessed with the help of a speculum and secretions were removed by swab. An endocervical brush was inserted, gently pressed against the wall of the cervix, rotated 5 times clockwise, and removed from the endocervical canal. The brush was then placed into a SurePath vial and refrigerated at 4-6°C until DNA extraction.

### 2.4. HPV detection

From the liquid-based sample, an aliquot was made in a laminar flow hood for the identification of HPV, by broad spectrum genotyping, using the 37 HPV GenoArray Diagnostic Kit (Hybribio® Diagnostics Ltd., Sheung Wan, Hong Kong). The kit detected the presence of 37 different HPV genotypes: 15 high (16, 18, 31, 33, 35, 39, 45, 51, 52, 53, 56, 58, 59, 66, 68), 6 low (6, 11, 42, 43, 44, 81 (CP8304)) and 16 undetermined risk (26, 34, 40, 54, 55, 57, 61, 67, 69, 70, 71, 72, 73, 82, 83, 84). HPV DNA was extracted from cervical cells using an alkaline lysis method. Samples were centrifuged for 10 min at 14000 rpm at 20°C, resuspended in 100 μL elution buffer, and stored at −20°C until PCR. The concentration and purity of DNA (OD260/OD280 1.6–1.8) was determined by Nanodrop 2000 (Thermo Fisher Scientific, CA, United States). This was followed by amplification, according to manufacturer recommendations, through conventional PCR and utilization of biotinylated PGMY primers (Thermocycler Applied Biosystems® /GeneAmp® 9,700). The PCR mix was prepared according to the manufacturer’s instructions to obtain a final reaction volume of 25 μL (23.25 μL PCR Mix, 0.75 μL of DNA Taq polymerase 5 U/μL and 1 μL of DNA). Amplification was performed with an initial denaturation at 95°C for 5 min (min), followed by 40 cycles of denaturation at 95°C for 20 s (s), annealing at 55°C for 30 s, and elongation at 72°C for 30 s, then a final elongation at 72°C for 5 min.

**Figure 1:**
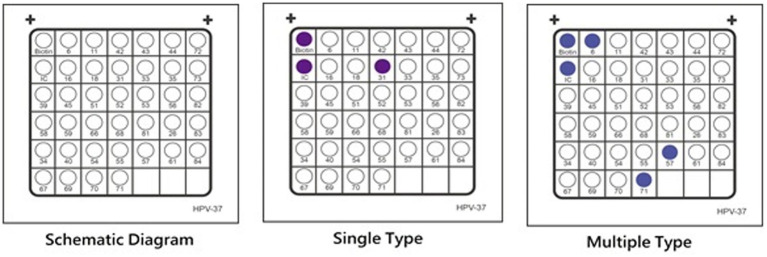
Schematic Diagram for HPV Genotyping Results: Retrieved from Hybridbio manufacturing information.

After amplification, the genetic material was denatured and subjected to membrane hybridization for 20 min using specific probes. All assays included a positive and a negative PCR control in addition to the hybridization control itself. The hybridization process was carried out using the HibriMax (Hybribio) kit according to the manufacturer instructions. This was conducted by washing, addition of streptavidin–horseradish peroxidase conjugate, membrane blocking, and addition of developer substrate (nitroblue tetrazolium-5-bromo-4-chloro3-indolyl phosphate). The membranes contained the immobilized probes of the genotypes of interest that hybridize with biotinylated amplified PCR products. Streptavidin–horseradish peroxidase conjugate was added to bind to the biotinylated PCR products. The direct visualization of the breakdown product (purple precipitate) from the addition of substrate nitroblue tetrazolium-5-bromo-4-chloro3-indolyl phosphate, was interpreted as positive for the corresponding HPV DNA type as indicated in the schematic diagram (Figure 1).

### 2.5. Statistical processing

Data was stored in an electronic database and processed using IBM SPSS 23.0 statistical software. Analysis of frequency and measures of central tendency position (mean, confidence intervals, percentiles) and dispersion (standard deviation, range) were used. Differences between groups were established using the Mann–Whitney or Kruskall-Wallis *U* test for determining whether sets are from the same distribution. Differences in proportions and bivariate association were estimated using cross tables (Z test for comparison of proportions by columns, *X^2^* test, Cramer’s V). The risk estimation used odds ratio (OR) with confidence intervals from bivariate analysis and confirmed by binary logistic regression using the progressive Wald method. The significance level of all tests was less than or equal to 0.05. The equation that describes HPV infection probability derives from a mathematical model of binomial logistic regression.

$$p\left(x\right)=\frac{1}{1+e^{-\left(\beta_{0}+\beta_{1}x\right)}}$$

### 2.6. Ethical aspects

The research protocol ensured informed consent and good practice established in the Declaration of Helsinki. Prior consent from all subjects involved was obtained by signature after the research objectives were presented orally and in writing. The anonymity and the will of the respondents and interviewees regarding the disclosure of information was respected. The research was reviewed and approved by the Catholic University of Cuenca Human Research Ethics Committee with resolution number CEISH 2018–09-017 R.

## 3. Results

### 3.1. Sociodemographic characterization

Sociodemographic results of the survey are shown in Table 1.

**Table 1:** Sociodemographic variables of women in Cuenca-Ecuador, 2019 *N* = 120.

Variables	Categories	Frequency	Percentage %	*X*^2^; *p*
Biological age	18–30	48	40.0	*X^2^* = 73.876; *p* = 0.000
	31–45	50	41.7	
	46–55	14	11.7	
	56–65	8	6.7	
Place of residence	Urban	73	60.8	*X^2^* = 5.633; *p* = 0.018
	Rural	47	39.2	
Education level	None-elementary	15	12.5	*X^2^* = 79.933; *p* = 0.000
	High school-college	105	87.5	
Marital status	Single	42	35.0	*X^2^* = 40.467; *p* = 0.000
	Divorce	15	12.5	
	Married	52	43.3	
	Join in law	11	9.2	
Personal incomes (USD/month)	$522 or less	69	57.5	*X^2^* = 83.119; *p* = 0.000
	$523–$874	25	20.8	
	$875–$1,200	15	12.5	
	More than $1,200	11	9.2	
Alcohol consumption (current or past)	Consumption under 10 beers or 5 glass of wine, or a bottle of rum per week	43	35.8	*X^2^* = 63.050; *p* = 0.000
	Consumption over 10 beers or 5 glass of wine, or a bottle of rum per week	77	64.2	
Tobacco consumption (current or past)	Under 100 cigarettes in your life	92	76.7	*X^2^* = 34.133; *p* = 0.000
	Over 100 cigarettes in your life	28	23.3	
Use hard drugs (current or past)	No	113	94.2	*X^2^* = 96.633; *p* = 0.000
	Yes	7	5.8	
Age of first coital intercourse	18 or younger	43	35.8	*X^2^* = 9.633; *p* = 0.002
	19 years or older	77	64.2	
Number of sexual partners	1 or 2	93	77.5	*X^2^* = 36.300; *p* = 0.000
	3 or 4	20	16.7	
	5 or more	7	5.8	
Frequency intercourse per month	1–2 / month	43	35.8	*X^2^* = 54.600; *p* = 0.000
	3–4 / month	28	23.3	
	5–7 / month	24	20.0	
	8–11 / month	15	12.5	
	More than 11/ month	7	5.8	
	Daily	3	2.5	
Use of condom during intercourse	Always or often	23	19.2	*X^2^* = 34.133; *p* = 0.000
	Sometimes or never	97	80.8	
Access to gynecologic care	At least once per year	102	85.0	*X^2^* = 58.800; *p* = 0.000
	Less than one per year	18	15.0	
Parity	One or less full pregnancy	68	56.7	*X^2^* = 2.133; *p* = 0.144
	Two or more full pregnancy	52	43.3	
History of STI	None	9	7.5	*X^2^* = 10.420; *p* = 0.002
	I do not know	28	23.3	
	Genital herpes	1	0.8	
	HIV/AIDS	1	0.8	
	Trichomoniasis	3	2.5	
	Chlamydia	77	64.2	
	Several ITS_s_	1	0.8	
Contraception use	None	46	38.3	*X^2^* = 50.550; *p* = 0.000
	Mainly condom	23	19.2	
	Pills	45	37.5	
	IUD	6	5.0	
Immunosuppression	Yes	84	70.8	*X^2^* = 19.200; *p* = 0.000
	No	36	29.2	
Knowledge of HPV (scale of 20)	X̄ = 13.57; SD = 2.01; IC 95% =13.21–13.94			
Immunosuppression symptoms (scale of 10)	X̄ = 7.52 SD = 3.76 IC 95% = 6.84–8.20			

The average age of participants was 35.6 years old (+/− 10.9). Most patients were between 31–45 years (41.7%), and 18–30 years (40.0% of patients) followed by ages 46–55 (11.7%) and 56–65 (6.6%) (*X^2^* = 73.876; *p* = 0.000). The place of residence was predominantly urban (60.8%) over rural areas (39.2%) (*X^2^* = 5.633; *p* = 0.018). Must participants had a secondary or university level of education (87.5%; *X^2^* = 79.933; *p* = 0.000). Additional demographics revealed that 78.3% of the sample had incomes below $875 USD (*X^2^* = 83.119; *p* = 0.000) 64.2% consumed alcohol regularly (*X^2^* = 63.050; *p* = 0.000), 23.3% of participants had a current or past consumption of tobacco (*X^2^* = 34.133; *p* = 0.000), and only 5.8% of participants have consumed hard drugs (*X^2^* = 93.633; *p* = 0.000).

Most participants (52.5%) had a partner (43.3% married, 9.2% common law). The remaining 47.5% were either single (35.0%) or divorced (12.50%) (*X^2^* = 40.467; *p* = 0.000). For a majority of the sample, the first instance of coitarche occurred at age 19 years or older (64.2%) (*X^2^* = 9.633; *p* = 0.002). The number of sexual partners per month varied throughout the participants with 77.5% indicating they have had 1–2 sexual partners, 16.7% between 3–4 partners, and 5.8% 5 or more partners (*X^2^* = 36.300; *p* = 0.000). 59.1% of sample patients reported a frequency of 1–4 sexual relations per month (*X^2^* = 54.600; *p* = 0.000).

Frequency of condom use in sexual intercourse was in low proportion (19.2%) compared to women that never or sometimes use condoms in coital (80.8%) (*X^2^* = 34.133; *p* = 0.000). 42.5% of the participants were currently using oral contraceptives or intrauterine devices and 38.3% did not use any contraceptive method (*X^2^* = 50.550; *p* = 0.000). The majority of women had given birth to one or no children (56.7%) and 43.3% were multiparous (*X^2^* = 2.133; *p* = 0.144). 69.2% of participants reported having a history of STIs (besides HPV) as opposed to 7.5% never having contracted an STI. It should be noted that 23.3% were unaware of their STI history (*X^2^* = 10.420; *p* = 0.002).

The access to gynecological health care had high occurrence with 85.0% of the sample population having at least one appointment per year, and the remaining participants (15.0%) having less than one appointment per year (*X^2^* = 58.800; *p* = 0.000). Clinical immunosuppression was found in 70.8% of the women (*X^2^* = 19.200; *p* = 0.000).

The sample population had an average level of knowledge on HPV of 13.6 +/− 2.0 (95% CI 13.2–13.9) on a scale of 20. There were significant differences in knowledge according to the level of education (*U* = 425.5; *p* = 0.004). Background knowledge on HPV was higher for the group with high school-college education compared to elementary-no education. No differences in knowledge were found due to age (*U* = 1442.0; *p* = 0.150) or marital status (*U* = 1450.0; *p* = 0.122).

**Figure 2:**
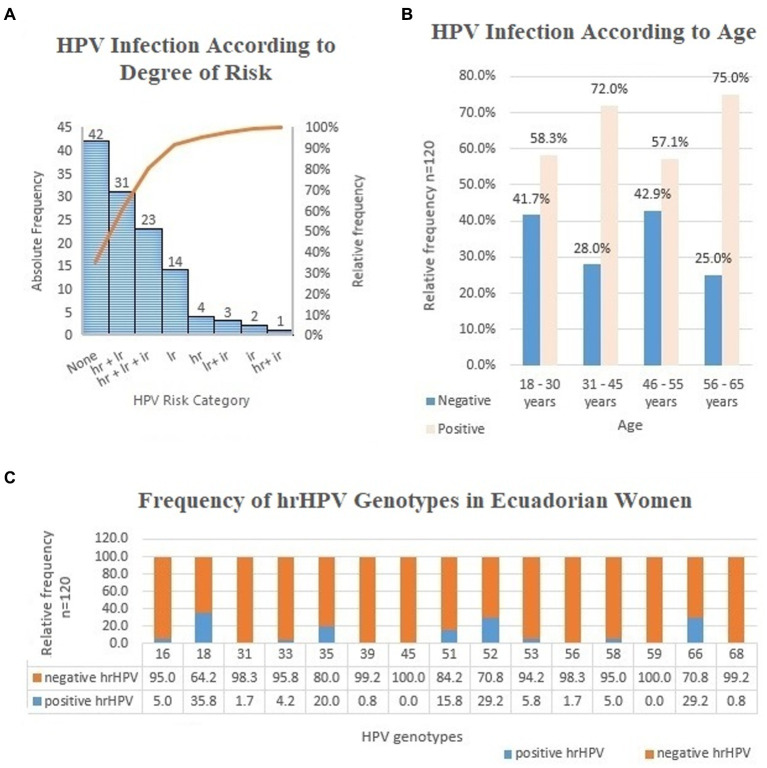
**(A)** Frequency analysis of HPV infection in women according to degree of risk, Cuenca, Ecuador; 2019. n=120 lr (low risk)-HPV 6, 11, 42, 43, 44, 81 (CP8304). ir (undetermined risk)-HPV 26, 34, 40, 54, 55, 57, 61, 67, 69, 70, 71, 72, 73, 82, 83, 84. hr (high risk)-HPV 16, 18, 31, 33, 35, 39, 45, 51, 52, 53, 56, 58, 59, 66, 68. **(B)** Frequency analysis of HPV infection in women according to age; Cuenca, Ecuador; 2019. *n*=120. **(C)** Frequency analysis of hr HPV genotypes in women; Cuenca, Ecuador; 2019. *n*=120.

The frequency of infection according to HPV strain is shown in Figure 2A. Of the total sample population, 65.0% were identified as HPV positive with 49.1% of the population identified to be infected with high-risk HPV. For the group of patients with HPV, 75.6% of infections corresponded to a high-risk genotype and 74.3% of the patients were identified to have coinfections with multiple HPV strains (48.3% of total sample population). Some coinfected women presented up to 8 different genotypes.

Even though the groups of women aged 56–65 and 31–45 years old had a higher proportion of HPV infections (Figure 2B), the Z test comparison for proportion was unable to establish significant differences between age groups (*p* > 0.050). There was no association between age groups and HPV infection (*X^2^* = 2.746; *p* = 0.432).

The frequency distribution of the hrHPV strains is shown in Figure 2C. The most frequent genotype was hrHPV 18, followed by genotypes 52, 66, 35, and 51 (*X^2^* = 7.234; *p* = 0.041). HPV 16 had a low frequency of infection in the group studied.

### 3.2. Modeling of HPV infection

The correlation analysis showed a moderate association between HPV infection and certain biological, sociodemographic, sexual and reproductive health variables. Table 2 shows the statistics for each of these associations.

**Table 2:** HPV infection according to Parity, Tobacco consumption, Number of sexual partners, Contraception and Immunosuppression symptoms in women from Cuenca Ecuador, 2019.

Variable	Categories	Frequency	HPV Infection		Total	*X^2^*, *p* Cramer’s V, *p*
			Negative	Positive		
Parity	0–1 full pregnancy	Counting	29_a_	39_b_	68	*X^2^* = 4.634; *p* = 0.045 Cramer’s V = 0.184 *p* = 0.041
		%	69.0	50.0	56.7	
	2 or more full pregnancy	Counting	13_a_	39_b_	52	
		%	31.0	50.0	43.3	
Tobacco consumption	No	Counting	37_a_	55_b_	92	*X^2^* = 4.718; *p* = 0.041 Cramer’s V = 0.198 *p* = 0.030
		%	88.1	70.5	76.7	
	Yes	Counting	5_a_	23_b_	28	
		%	11.9	29.5	23.3	
Number of sexual partners	1–2	Counting	37_a_	56_b_	93	*X^2^* = 4.160; *p* = 0.045 Cramer’s V = 0.186 *p* = 0.045
		%	88.1	71.8	77.5	
	3 or more	Counting	5_a_	22_b_	27	
		%	11.9	28.2	22.5	
Immunosuppression symptoms	No	Counting	20_a_	16_b_	36	*X^2^* = 9.552; *p* = 0.003 Cramer’s V = 0.282 *p* = 0.020
		%	47.6	20.5	30.0	
	Yes	Counting	22_a_	62_b_	84	
		%	52.4	79.5	70.0	
Use of oral contraceptive or IUD	No	Counting	37_a_	32_b_	69	*X^2^* = 24.751; *p* = 0.000 Cramer’s V = 0.454 *p* = 0.000
		%	88.1	41.0	57.5	
	Yes	Counting	5_a_	46_b_	51	
		%	11.9	59.0	42.5	
Total		Counting	42	78	120	
		%	100	100	100	

Each letter of the subscript denotes a subset of the category whose column proportions do not differ significantly from each other at the 0.050 level. HPV Infection (Yes/No) x Parity (2 or more full pregnancy) (Yes). OR = 2.231; CI 95% 1.012–4.918. HPV Infection (Yes/No) x Tobacco consumption (Yes). OR = 3.095; CI 95% 1.080–8.870. HPV Infection (Yes/No) x Number of sexual partners (3 or more) (Yes). OR = 2.907; CI 95% 1.011–8.358. HPV Infection (Yes/No) X Immunosuppression symptoms (Yes). OR = 3.523; CI 95% 1.555–7.980. HPV Infection (Yes/No) X Use of oral contraceptive or IUD (Yes). OR = 10.638; CI 95% 3.771–30.010.

While overall no association was found between HPV infection and condom use, when accounting for marital status, participants who were single or in common law unions, condom use was associated with lower infectivity (Fisher’s *X^2^* = 5.852; *p* = 0.026; V Cramer = − 0.293; *p* = 0.016). For married women, this association was not found (*X^2^* = 1.231; *p* = 0.412).

The remaining variables included: the age at which the participant became sexually active (*X^2^* = 3,234; *p* = 0.357), knowledge about HPV (*X^2^* = 0.261; *p* = 0.610), gynecological medical care (*X^2^* = 0.141; *p* = 0.708), place of residence (*X^2^* = 0.369; *p* = 0.562), education level (*X^2^* = 0.188; *p* = 0.664), alcohol consumption (*X^2^* = 0.669; *p* = 0.413), age groups (*X^2^* = 2.033; *p* = 0.362), previous STIs (*X^2^* = 1.553; *p* = 0.276), frequency of sexual intercourse (*X^2^* = 0.701; *p* = 0.441), and marital status (*X*^2^ = 1.528; *p* = 0.250). These were not associated with HPV infection in the sample studied.

Binary logistic regression confirmed an association between parity, immunosuppression symptoms, use of oral contraceptives/IUDs, and positive HPV infection. The rest of the variables were excluded due to their low statistical significance (*p* > 0.050) or low predictive power with a non-significant OR. No confounding or interaction variables were found. The characteristics of the model obtained by binary logistic regression are shown in Table 3.

**Table 3:** Explanatory model of HPV infection through health and sociodemographic variables.

Step 1^a^	B	Standard error	Wald	Sig.	Exp(B)	95% CI. for EXP(B)	
						Lower	Upper
Immunosuppression symptoms (Yes = 1)	0.979	0.502	3.796	0.050	2.661	1.094	7.120
Contraception (Yes = 1)	2.584	0.596	18.787	0.000	13.254	4.119	42.646
Multiparity (Yes = 1)	1.531	0.516	8.812	0.003	4.622	1.682	12.699
Tobacco consumption (Yes = 1)	0.719	0.666	1.165	0.280	2.051	0.556	7.564
Number of sexual partners (Yes = 1)	0.648	0.689	0.884	0.347	1.912	0.495	7.385
Constant	−1.755	0.523	11.259	0.001	0.173		

Immunosuppression symptoms (1) - High frequency of infectious diseases symptoms. Score > 5 in scale. Contraception (1) - Use of oral contraceptive or IUD. Multiparity (1) - 2 or more full pregnancy. Tobacco consumption (1) - Consumption of more than 100 cigarettes in the past or present. Number of sexual partners (1) - 3 or more sexual partners. ^a^Backward Elimination Method (Wald).

The model has significant values of Hosmer Lemeshow test (*X^2^* = 6.698; fg = 4; *p* = 0.845), diagnostic power of 81.7%, a high sensitivity of 89.5% and an acceptable specificity of 73.8%. This model makes it possible to explain up to 42.9% of the variance of the infection with HPV according to the Nagelkerke coefficient of Determination (*R*^2^ = 0.429). Even though the model is not comprehensively adequate, select variables have been viewed as possible explanations for HPV infection. This model attempts to rule out tobacco use and number of sexual partners as significant explanations of infection according to their respective OR values.

The equation that describes HPV infection probability is as follows:

$$\begin{array}{l} p\left(HPV\;Infection\right)\\ \;\;\;\;\;\;=1/\left(1+e^{\left(1.755-2.584*Contraception-1.531*Parity-0.979*Immunosuppression\right)}\right) \end{array}$$

## 4. Discussion

The World Health Organization (WHO) reports more than 1 million new sexually transmitted infections each day (5). The frequency of infection among women is found to be higher in Latin America (16%) than more developed areas, such as North America (4.7%) (2). The most prevalent viral types contributing to cervical cancer burden in Latin America are HPV 16, 18, 31, 58, 33, 45, and 52 in decreasing frequency.

In Ecuador, there is no epidemiological study about HPV infection within the country in its entirety. For instance, ENSANUT’s 2019 report (National Survey for Health and Nutrition) does not contain information about HPV infection. Neither exists a national program to test HPV infection, even when deaths for UCC increased to 902 women in the country in 2019 (19).

A 2016 study found that in a cohort of 164 Ecuadorian women, 86% tested positive for HPV. Out of those who were tested positive, the most common strains found were HPV 16 at 42% and HPV 58 at 31%. HPV18 was only detected in 3% of the samples (20). Comparatively in Ecuador, prevalence by strain contributing to invasive cervical cancer ranks HPV 16, 58, 52, 31, 59, 39 in decreasing frequency (21). Another study carried out in Cañar, a region of the Ecuadorian highlands, confirms a shift in infection patterns towards hrHPV 31, 58, 59, and 66 in around 20% of the infected women (22). A study conducted in the city of Guayaquil between 2015–2018 in 800 participants from the Ecuadorian coast found a high percentage of infection by non 16–18 HPV strains in men and women (51.38%) (23). The same change of HPV patron infection is shown in Cuenca where the most prevalent HPV strains were 58, 51, 31, 52 and 53 (24).

A systematic review published in 2021 identified the main results of the HPV investigations in the country in the last 7 years (25, 26). These articles have a few differences with the present investigation: an increase in hrHPV 18 and hrHPV31 infections, and a low level of hrHPV-16. Other systematic reviews also show heterogeneity in the circulating strains according to the country’s region. In the 2021 report from the HPV Information Center, HPV strains 16 (38%), 58 (28%), 52 (12%), and 31 (10%) were found to be the most frequent strains in Ecuadorian women with invasive cervical cancer by histology. Notably, HPV18 was not listed in the top ten most frequent strains (21). These findings highlight high rates of infection in the Ecuadorian highlands region. Results were heterogeneous in populations, sampling, and HPV identification techniques. Differences in current studies hinder a thorough comparison needed to achieve agreement and generalizability for HPV trends in Ecuador.

The presence of different strains infecting women according to the geographic region in Ecuador may be related to population characteristics (biological, cultural, and social) which suggest differences in susceptibility to HPV infection. Biological characteristics of the host, such as immune status, trophic predisposition to the virus, and favorable vaginal biome could promote a greater viral infection.

Although infection with HPV is a high-frequency phenomenon, viral clearance occurs in 80–90% of infected women (27). One of the main factors in blocking or eliminating infection is the functioning of the host’s immune system. Immunological status of the patient is relevant in the infection and persistence of HPV (28). Investigations have found a protective humoral response against the L1 and L2 proteins present in the viral capsid and the presence of non-neutralizing antibodies against the early phase proteins E6 and E7 (29). Other factors related to the persistence of the infection are the APOBEC3A proteins, which act as antiviral protection mechanisms through epigenetic mechanisms (30). It suggests that innate defense could be a relevant mechanism to destroy virus infection of mucosal (31). Vaccination against HPV has also been able to generate protection against infection in numerous populations (32), confirming the role of the immune system in preventing or eliminating HPV infection (28). Currently, there are no scientific reports that establish the efficacy and type of immune response after HPV vaccination in Ecuador.

The substantial differences in the circulating genotypes in the south highlands of Ecuador and a possible change in the patterns of infection with HPV suggest a specific approach in diagnostic research, prevention, and treatment (33, 34). Non-cytology-based approaches to diagnosis could have high efficacy in low-resource settings and can decrease the prevalence of pre-cervical cancer lesions (35). A national program for HPV infection diagnosis could unify the diverse diagnostic methods used in clinical laboratories of the country, and generate more reliable data for decision-making in HPV strategy.

The age distribution of HPV infection did not show a classical “*U*” shape view like in several Latin-American countries (Brazil, Costa Rica, México, and Chile) (36). In these countries, HPV infection was more widespread in older women. Conversely, our study found that young women less than 30 years old had a similar proportion of HPV infection to the group of 31–45 years old. This fact suggests different patterns of population infection, differential effectiveness of vaccination campaigns realized in 2014 (38), or a possible bias due to the small sample for investigation.

The role of risky sexual behavior as a determinant of STIs has been validated by numerous investigations (37–41). Unlike reports in the literature (42), our study shows that the age of first coitarche and the number of sexual partners is not associated with STIs.

There is also a lack of overall correlation between condom use and HPV infection. This finding may be related to the inconsistent use of condoms in married women who are infected by marital and extramarital sex, which is identified by Calatrava et al. in other populations of women infected with STIs (37).

Although tobacco use potentiates the appearance of cervical cancer in women infected with hrHPV (11), the effect on HPV infection is not well understood. Epidemiological research made by Utami et al. fails to prove the statistical association between HPV infection and tobacco use in cross-sectional research (43). HPV could be a synergic or potentiating factor for HPV infection. Subsequent investigations could shed light on the effect of tobacco use and HPV infection by controlling the level and frequency of tobacco consumption in a prospective cohort’s design.

Hormonal contraception and HPV infection have been found to induce the progression of UCC (44). Estrogens that can be found within these contraceptives are associated with the development of UCC in conjunction with hrHPV. However, other studies have found no significant association between hormonal contraception and cervical cancer when controlling for HPV infection (45) or are inconclusive about HPV infection risk (46).

Previous research has corroborated an association between the use of estrogenic oral contraceptives and hrHPV infection. The influence of female hormones on HPV infection is not well understood, but the effect has recently been suggested. Estrogens induce the expression of the polysaccharide layer present in the basement membrane of the cervical epithelium of animal models (47, 48). Researchers describe that the mechanism of entry of HPV into cells occurs through the binding of the viral L1 protein (49) to proteoglycans (heparan sulfate) (50) present in the basement membrane of the epithelium of animal models. Estrogen receptor knockout mice have shown protection against viral infection of the reproductive system (51). Recent research has also found an association between estrogenic oral contraceptives and hrHPV infectivity (52). Hormonal estrogen contraception could enhance the entrance of HPV through the high level of heparan sulfate in the epithelial cell surface (53).

Although previous epidemiological studies documented parity as a risk factor for cervical cancer, the reported strength of association is variable and inconsistent. Several investigations revealed a connection between parity, HPV infection, and CCU (54). These studies suggest that a high rate of cervical abnormalities during pregnancy is conducive to cervical lesions and make epithelial cells more susceptible to HPV infection. The main problem is determining the parity cut-off due to differing values between studies.

### 4.1. Study limitations

Interpretations of the discussed findings should consider the tested demographic in relation to sampling method and sociocultural characteristics. The extrapolation of results must be assessed carefully in different contexts. While utilizing the random selection method, there was potential for bias in sampling. Participants who came for healthcare services at the facility expressed interest in research participation, and this may introduce potential bias with its convenience.

Sample size and cultural diversity of participants was considered a limitation of this study. We acknowledge that the results represent a small-scale sample of the sexually active female population of the Ecuadorian highlands. Further studies should be carried out to increase external validity. We suggest continuing with different multicentric studies to substantiate diagnostic value within a large and diverse group of women in regions of Ecuador.

The survey method can also be considered a limitation based on the participant’s ability to answer accurately. To achieve reliable data, a comfortable environment must be established to maintain confidence in doctor-patient disclosure. Although a relationship was found between HPV and immunosuppression symptoms, it is inaccurate to draw a correlation based on our study due to the reliability of the survey method and absence of molecular testing. These clinical implications could also be confounded by participants, resulting in a source of perception bias. We suggest that our method should be validated further through pilot testing in populations under study.

## 5. Conclusion

The predominant strains of HPV among Ecuadorian women are diverse. The risk of HPV infection is a complex phenomenon where biological and psychosocial variables are integrated into a model. HPV pre-screening in populations with limited access to health services, low socioeconomic status, and negative sociocultural beliefs about STIs could use surveys as a step before screening for HPV infections. The diagnostic value of the model should be tested further in multicenter studies all over Ecuador.

## Data availability statement

The datasets presented in this study can be found in online repositories. The names of the repository/repositories and accession number(s) can be found at: https://ucacueedu-my.sharepoint.com/:f:/g/personal/jbaculima_ucacue_edu_ec/ElO4GinuTYpIqSv39NWCDI8B4d_LBdfiiruxKz4W2mU1pw?e=5KZNNhAccesionkey:Investigacion.BF.

## Ethics statement

The studies involving human participants were reviewed and approved by Human Research Ethics Committee UCACUE. The patients/participants provided their written informed consent to participate in this study.

## Author contributions

CR: research design, survey design and validation, biospecimen processing, and data processing and statistical analysis. YH: research design, survey design and validation, and data processing and statistical analysis. DA: research design and data processing and statistical analysis. ZS and LM: data processing and statistical analysis, biospecimen collection, and sociodemographic data collection. EC, LG, and MM: survey design and validation, biospecimen processing, and sociodemographic data collection. MS: biospecimen collection and sociodemographic data collection. JB: data processing and statistical analysis. All authors contributed to the article and approved the submitted version.

## Funding

Funding was received from UCACUE (Grant UCACUE-DIPVP-2018-165-OF).

## Conflict of interest

CR and JB were employed by Diagnostic Department, MEDsan, Inc., Saint Petersburg, FL, United States.

The remaining authors declare that the research was conducted in the absence of any commercial or financial relationships that could be construed as a potential conflict of interest.

## Publisher’s note

All claims expressed in this article are solely those of the authors and do not necessarily represent those of their affiliated organizations, or those of the publisher, the editors and the reviewers. Any product that may be evaluated in this article, or claim that may be made by its manufacturer, is not guaranteed or endorsed by the publisher.
